# Inadequate Sensitivity of Laboratory Risk Indicator to Rule Out Necrotizing Fasciitis in the Emergency Department

**DOI:** 10.5811/westjem.2016.2.29069

**Published:** 2016-04-26

**Authors:** Elizabeth Burner, Sean O. Henderson, Guenevere Burke, Jeffrey Nakashioya, Jerome R. Hoffman

**Affiliations:** * Keck School of Medicine of the University of Southern California, Department of Emergency Medicine, Los Angeles, California; † George Washington University, Department of Emergency Medicine, Washington, DC; ‡ University of California Los Angeles, Department of Emergency Medicine, Los Angeles, California

## Abstract

**Introduction:**

Necrotizing fasciitis (NF) is a life-threatening illness, particularly when surgical debridement is delayed. The Laboratory Risk Indicator for Necrotizing Fasciitis (LRINEC) score was developed to identify patients at higher risk for NF. Despite limited information in this regard, the LRINEC score is often used to “rule out” NF if negative. We describe the sensitivity of the LRINEC score in emergency department (ED) patients for the diagnosis of NF.

**Methods:**

We conducted a chart review of ED patients in whom coding of hospital discharge diagnoses included NF. We employed standard methods to minimize bias. We used laboratory data to calculate the LRINEC score, and confirmed the diagnosis of NF via explicit chart review. We then calculated the sensitivity of a positive LRINEC score (standardly defined as six or greater) in our cohort. We examined the role of patient characteristics in the performance of the LRINEC score. Finally, we performed sensitivity analyses to estimate whether missing data for c-reactive protein (CRP) results were likely to impact our results.

**Results:**

Of 266 ED patients coded as having a discharge diagnosis of NF, we were able to confirm the diagnosis, by chart review, in 167. We were able to calculate a LRINEC score in only 80 patients (due to absence of an initial CRP value); an LRINEC score of 6 or greater had a sensitivity of 77%. Sensitivity analyses of missing data supported our finding of inadequate sensitivity to rule out NF. In sub-analysis, NF patients with concurrent diabetes were more likely to be accurately categorized by the LRINEC score.

**Conclusion:**

Used in isolation, the LRINEC score is not sufficiently sensitive to rule out NF in a general ED population.

## INTRODUCTION

Necrotizing fasciitis (NF) is a life-threatening infection with high mortality. Because NF can be misdiagnosed as a less lethal mimic, such as cellulitis and abscess, efforts have been made to identify clinical features that could help clinicians accurately diagnose NF and avoid delays to surgical debridement.^1^ Prior retrospective studies have shown certain laboratory values, particularly an extremely elevated leukocyte count and a low sodium concentration, are associated with NF.^2^ These abnormal values might help clinicians distinguish NF from less severe soft-tissue infections. The Laboratory Risk Indicator for Necrotizing Fasciitis (LRINEC) score was developed in a large cohort of admitted patients to identify patients at higher risk for NF.^3^ Patients are assigned a LRINEC score based on serum sodium, glucose, creatinine, c-reactive protein (CRP), leukocyte count and hemoglobin. Scores range from 0 to 13; a score 6 or greater was associated with a high risk of NF, and a score of 8 or greater with a very high risk.

The LRINEC score can be easily misapplied, however. The score was not designed to exclude NF in patients with a low-risk score, and case reports and small studies externally validating the score failed to replicate the high sensitivity and negative predictive value reported in the initial paper.^4^–^8^ Additionally, the sensitivity of the score has not been addressed specifically among emergency department (ED) patients.

We conducted this retrospective study to determine the sensitivity of the LRINEC score in ED patients with a confirmed diagnosis of NF and examine the role of patient factors in the score’s sensitivity. We also performed secondary analyses to estimate how missing laboratory values impacted our results.

## METHODS

The study was approved by the institutional review board prior to initiation.

The study cohort consisted of patients evaluated in the ED of Los Angeles County+University of Southern California Medical Center (LAC+USC) with NF. The LAC+USC ED is an urban, academic, tertiary care hospital. Patients were identified by search of all International Statistical Classification of Diseases and Related Health Problems, Ninth Revision (ICD-9) hospital discharge codes between April 2003 and April 2013; charts of all patients coded as having NF (728.86) were then reviewed, and further categorized as either “confirmed” or “unconfirmed” (see below.) Patients who were not initially evaluated in the ED or who developed NF post-operatively after admission were excluded.

### Primary Study: Sensitivity of the LRINEC Score in ED Patients

#### Chart Abstraction Methods

We used standard abstraction methods to minimize bias.^9^ All abstractors received training. Medical students blinded to the study hypothesis calculated the LRINEC score after two training sessions; the lead author also reviewed their first results to ensure reliability and accuracy, and a coding guide was made available to abstractors (Appendix 1). The lead author reviewed 10% of cases, and with a Kappa calculated for abstracted variables.

#### Diagnosis Confirmation

The ICD-9 diagnosis of NF was confirmed if any of the following criteria were met: 1) NF was a diagnosis on the hospital discharge or death summary; 2) NF was confirmed at surgery, as documented by operative report; or 3) fascial necrosis was documented on an anatomic pathology specimen. Patients with ICD-9 coding of “NF” but in whom none of these criteria were met, were classified as “unconfirmed” and were excluded.

#### LRINEC Scores

During laboratory value review, abstractors were blinded to the final “confirmation” of NF in any individual case. Only the first value for each laboratory test was collected, eliminating conflicting data. Abstractors coded a result as “missing” if there was no result in the first 48 hours of the hospital course; this occurred only with CRP; all other laboratory tests were present for every patient. These cases were excluded as no score could be calculated.

#### Patient Characteristics

Patient age, gender and inpatient mortality were collected from administrative data. Past medical history was abstracted from medical history and final diagnoses on operative reports, discharge summaries and death notes.

### Primary Study: Sensitivity of LRINEC score, and Association with Patient Factors

A LRINEC score was calculated for each patient (Figure), and the overall sensitivity of the score was calculated using STATA version13, with a binomial model for confidence intervals. We included only patients with complete data. We used chi-squared tests and t-tests to examine the sensitivity of the LRINEC score when stratifying by patient factors of age, gender, inpatient mortality and history of cirrhosis, intravenous drug use or diabetes, known risk factors for developing NF.

### Secondary Study: Analysis of Missing Data

All cases without laboratory results to calculate a LRINEC score were missing a CRP, so we performed sensitivity analyses to determine how this impacted our results. We calculated a LRINEC score for each patient with missing data based on the assumption that the missing CRP value “would have been” positive in 50%, 77% (the sensitivity of the LRINEC score found in our cohort), or 100% of cases. Because of the large contribution of CRP to the LRINEC score, we assumed that if the CRP was positive, the LRINEC score would also be positive. We calculated the sensitivity of the score under at each of these assumptions for CRP.

## RESULTS

### Primary Study

A computerized records search identified 316 patients between April 2003 and April 2013 with ICD-9 discharge diagnosis of NF. We excluded 47 cases that were not admitted through the ED and three cases that developed NF in the post-operative period while inpatient. NF was “confirmed” by chart review in 167 cases, with 100% interrater agreement (Kappa=1.0), but only 80 of these patients had a CRP documented in the first 48 hours of presentation. The interrater reliability for a positive LRINEC score was excellent (Kappa=0.91), as was that for history of diabetes (K=0.84). Interrater reliability for history of cirrhosis and intravenous drug use was good (K=0.78 for both). Demographic characteristics and percentage of cases with each LRINEC score are shown in Table.

In the final study cohort, the overall sensitivity of the LRINEC score was 77% (CI [66–85]). Patients with diabetes were more correctly categorized by the score than patients without diabetes (85% vs 67%, p=0.04). There was no difference in the score’s sensitivity when patients were stratified by age, gender, inpatient mortality nor history of cirrhosis or intravenous drug use.

### Secondary Analyses

If the CRP and resultant LRINEC score are assumed to be “positive” in 50%, 77% or 100% of cases missing data, the sensitivity would be 63% (CI [55–70]), 76% (CI [69–82]) or 89% (CI [83–93]), respectively. Our analysis of missing data gives a range of sensitivities of as low as 55% to as high as 93%.

## DISCUSSION

In our population of ED patients with NF, the LRINEC score had a measured sensitivity of 77%, substantially lower than the 91% reported by Wong.^3^ Our population differs from the Wong cohort in that our patients came exclusively from the ED and were younger, more frequently male and had a higher mortality rate. Our finding that the LRINEC score, applied in isolation, would miss over 20% of cases of NF is consistent with reports of sensitivities between 68% and 80% in smaller studies based in surgical referral centers.^6^–^8^,^10^

While our sensitivity analyses, based on realistic possibilities regarding missing CRP data, suggest that the sensitivity of the LRINEC score could range between 55% and 93%, it is likely that the true value is less than 80%. Our calculated sensitivity for LRINEC may be artificially high, since in clinical practice this score is often used to decide whether a patient needs further work up or surgical management. Patients with NF and a falsely negative LRINEC score are less likely to have the diagnosis confirmed through pathologic specimens (and would be missed by our study methodology).

## LIMITATIONS

The focus of this study is the sensitivity of the LRINEC score in ED patients of one hospital; the findings may have limited generalizability. The use of a single ICD-9 code may miss cases of NF due to miscoding. The validity of any chart review is threatened to the extent that it relies on data that is frequently missing, internally inconsistent, and/or poorly gathered. To minimize this, we employed standard retrospective chart review methodology. Reviewers were trained and were largely blinded to our study hypothesis and outcome data. We conducted duplicate review to assess reliability, used precise definition of both independent and dependent variables, and relied on only initial laboratory data to decrease inconsistency. We minimized issues with missing data, except with regard to CRP. To address the high number of missing CRP values, we performed sensitivity analyses covering reasonable assumptions about how the missing values might have affected our results. As no possible values for the missing data could have produced a high sensitivity of the LRINEC, our primary conclusion – that a normal LRINEC score should not by itself be used to rule out NF – remains qualitatively unchallenged regardless of what these missing CRP values might have been. Additionally, it is possible that spectrum bias is present, and that the LRINEC score performs better in the most severe cases; however, an ideal diagnostic adjunct would aid a clinician in identifying the correct diagnosis in subtle cases.

## CONCLUSION

In this cohort, the LRINEC score with the standard cut-off of six would miss over 20% of cases of NF. Our results suggest that clinicians must maintain a high index of suspicion, and avoid the trap of using a “normal” LRINEC score, in isolation, to dismiss the diagnosis. While a lower cut-off might improve the sensitivity, the accompanying cost to specificity is not knowable in this study.

## Supplementary Information



## Figures and Tables

**Figure:**
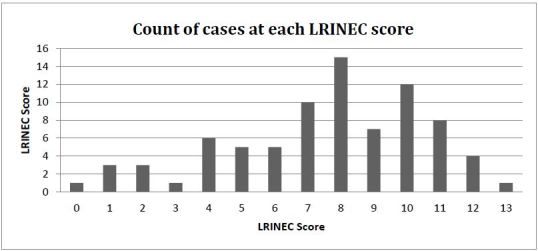
Count of cases at each Laboratory Risk Indicator for Necrotizing Fasciitis (LRINEC) score.

**Table:** Characteristics of patients with confirmed necrotizing fasciitis.

n=81	%
Male	80%
Inpatient mortality	35%
Age (mean, SD)	47.5 (1.4)
History of diabetes	49%
History IVDU	18%
History of cirrhosis	6%
LRINEC score positive	76%
LRINEC score negative	34%

*IVDU,* intravenous drug user; *LRINEC*, laboratory risk indicator for necrotizing fasciitis
