# Supplementary material for: Inadequate Sensitivity of Laboratory Risk Indicator to Rule Out Necrotizing Fasciitis in the Emergency Department
Source: West J Emerg Med. 2016 Apr 26;17(3):333–6. doi: 10.5811/westjem.2016.2.29069 (PMC4899066; doi:10.5811/westjem.2016.2.29069)
Supplement: Supplementary file 1 [file wjem-17-333-s001.pdf]

Necrotizing Fasciitis protocol

Laboratory variables:
from Affinity (Computerized results and record keeping)
Potassium
Sodium
Chloride
Bicarbonate
BUN
Creatinine
Glucose
Lactate
Creative protein (CRP)
White blood cell count
Neutrophil percent
% Bands
Hemoglobin
Hematocrit
Platelets
Erythrocyte sedimentation Rate (ESR)

Enter lab data

- Enter earliest value for that admission
- Only enter data that is within 48 hours of the first lab draw in time frame
- If a lab is not reported in the time frame, leave blank
- Click on Resulted Tests to double check that a lab is not available (especially CRP)
- Check the CRP and lactate units by clicking on the reported value: lab changes units sometimes. There are 10 deciliters in a liter, so if the lab reports 5 mg/dL, enter 50 mg/L
- If an "band estimate" is reported, report what is reported: <10 or the high end of the estimate for bands if a range is given, and the low end of estimated for platelets

Confirmation Necrotizing Fasciitis: From Affinity
Necrotizng fasciitis Surgical Path:
Check if there is surgical pathology available for the admission in question
If so, check for mention of necrosis of fascia, or for a pathologic diagnosis of necrotizing fasciitis. If either is mentioned, Yes for this variable.

Nec Fasc Op report
Check for operative reports for that admission. Look for operative findings of necrosis of fascia, or for a post-surgical diagnosis of necrotizing fasciitis. If either is mentioned, Yes for this variable.

Nec Fasc DC summary
Check discharge summary for that admission. Look for a discharge diagnosis of necrotizing fasciitis. If mentioned, yes for this variable.

Past medical history variables:
Diabetes in past medical history
DM op report
Check for operative reports for that admission. Look for preoperative or posteoperative diagnosis of diabetes. Check the patient presentation paragraph at beginning. Look for mention of diabetes. If either is mentioned, Yes for this variable.

DM DC summary
Check discharge summary for that admission. Look for a discharge diagnosis of diabetes. Check the patient presentation paragraph at beginning. Look for mention of diabetes. If either is mentioned, Yes for this variable.

Look at discharge medications. If metformin, glyburide, glipizide or insulin listed, yes for this variable.

Cirrhosis in past medical history
Cirrhosis Op report
Check for operative reports for that admission. Look for preoperative or posteoperative diagnosis of cirrhosis. Check the patient presentation paragraph at beginning. Look for mention of cirrhosis. If either is mentioned, Yes for this variable.

Cirrhosis DC summary
Check discharge summary for that admission. Look for a discharge diagnosis of cirrhosis. Check the patient presentation paragraph at beginning. Look for mention of cirrhosis. If either is mentioned, Yes for this variable.

IVDU in past medical history
IVDU Op report
Check for operative reports for that admission. Look for preoperative or posteoperative diagnosis of IVDU. Check the patient presentation paragraph at beginning. Look for mention of IVDU. If either is mentioned, Yes for this variable.

IVDU DC summary
Check discharge summary for that admission. Look for a discharge diagnosis of IVDU. Check the patient presentation paragraph at beginning. Look for mention of IVDU. If either is mentioned, Yes for this variable.

Microbiology variables: from Affinity
Use Microbiology View in Affinity
Limit window to one day before admission to 30 days after admission
Look for cultures with bacteria identified in the following order:
1. surgical pathology biopsy culture
2. fluid aspirate culture
3. wound culture
4. blood culture

If a bacterial is identified record the following on the data sheet
-Culture source
-Polymicrobial infection yes or no
-monomicrobial infection yes or no
-Group a strep Yes or No
-Group B strep Yes or No
-MRSA yes or no
-MSSA yes or no
-other bacteria identified: free text
